# White carbon: Fluorescent carbon nanoparticles with tunable quantum yield in a reproducible green synthesis

**DOI:** 10.1038/srep28557

**Published:** 2016-06-23

**Authors:** Till T. Meiling, Piotr J. Cywiński, Ilko Bald

**Affiliations:** 1 Physical Chemistry, Institute of Chemistry, University of Potsdam, 14476 Potsdam-Golm, Germany; 2 Functional Materials and Devices, Fraunhofer Institute for Applied Polymer Research, 14476 Potsdam-Golm, Germany; 3 Coordination Complexes and Functional Materials, Institute of Physical Chemistry, Polish Academy of Sciences, 01-224 Warsaw, Poland; 4 BAM, Federal Institute of Material Research and Testing, 12489 Berlin, Germany

## Abstract

In this study, a new reliable, economic, and environmentally-friendly one-step synthesis is established to obtain carbon nanodots (CNDs) with well-defined and reproducible photoluminescence (PL) properties via the microwave-assisted hydrothermal treatment of starch and Tris-acetate-EDTA (TAE) buffer as carbon sources. Three kinds of CNDs are prepared using different sets of above mentioned starting materials. The as-synthesized CNDs: C-CND (starch only), N-CND 1 (starch in TAE) and N-CND 2 (TAE only) exhibit highly homogenous PL and are ready to use without need for further purification. The CNDs are stable over a long period of time (>1 year) either in solution or as freeze-dried powder. Depending on starting material, CNDs with PL quantum yield (PLQY) ranging from less than 1% up to 28% are obtained. The influence of the precursor concentration, reaction time and type of additives on the optical properties (UV-Vis absorption, PL emission spectrum and PLQY) is carefully investigated, providing insight into the chemical processes that occur during CND formation. Remarkably, upon freeze-drying the initially brown CND-solution turns into a non-fluorescent white/slightly brown powder which recovers PL in aqueous solution and can potentially be applied as fluorescent marker in bio-imaging, as a reduction agent or as a photocatalyst.

Since their first description in 2006 carbon dots (CNDs) have attracted considerable attention of both fundamental research and industry^1^. Due to their promising properties CNDs have already been implemented in electrochemical applications^2^, waste water treatment^3^; (photo)catalysis^4^; bio-imaging^5^,^6^,^7^ and bio-technology as well as in chemical sensing^6^,^8^,^9^ and optoelectronic devices like LEDs^10^,^11^,^12^,^13^,^14^. Since carbon is the element that can be found in every organic matter, the CNDs can be easily prepared from readily accessible materials. However, the synthesis methods are typically expensive and depend on complex and time-consuming processes or severe synthesis conditions and toxic chemicals^5^,^6^,^7^,^11^,^15^. Typical CND preparation methods are arc-discharge^10^,^16^, laser ablation^1^,^17^, electrochemical synthesis^4^,^18^,^19^, organic pyrolysis^7^,^9^,^20^,^21^ or hydrothermal methods^6^,^8^,^22^. Therefore, to reduce the overall preparation costs and to make use of biological waste, natural carbon sources such as pomelo peal^8^, egg-white and egg-yolk^20^, orange juice^22^ and even eggshells^23^ have been used for the preparation of CNDs. While the use of waste is desirable, especially to avoid competition with essential food production^24^, most starting-materials used in those studies lack the essential purity and structural homogeneity to obtain homogenous CNDs. This is due to the fact that under corresponding treatment nearly any carbon-source will form CNDs.

Among various kinds of CNDs, nitrogen-doped carbon nanodots (N-CNDs) have emerged to be attractive materials due to their excellent photoluminescence (PL) properties^23^. However, most approaches reported to date have resulted in N-CNDs with heterogeneous PL properties, indefinite composition and they required extensive purification steps. For next generation luminescent probes the foreign atom doping of CNDs, especially with nitrogen, is of high interest, as it can drastically increase the overall PLQY^25^. This can be traced back to the fact that nitrogen has a comparable atomic size to carbon and five valence electrons to bind carbon atoms. In the presence of nitrogen, the electronic density of states is changed and/or emissive trap states for photo excited electrons are generated, which modify the band-gap energy thereby changing the optoelectronic properties of the CNDs^25^. In order to obtain homogeneous CNDs, a fundamental understanding of the CND formation mechanism, well-defined starting materials and deliberate use of additives are essential. A perfect carbon source for green CND synthesis should be soluble in water (green chemistry), accessible worldwide with defined and well-known properties, should not be in direct competition with essential food production, and last but not least, be cost-effective^10^,^26^,^27^. While the price of additives or carbon source plays a minor role in fundamental research, it may play a major role when commercial quantities are considered. Noticeably, the ease of the processing and no need for tedious and long purification has tremendous impact on the overall costs of the final material. Therefore, efforts have to be made to avoid today’s scenario observed for semiconductor quantum dots (QDs). While having amazing properties and interesting uses, they still haven’t achieved their full potential in commercial applications due to significant production costs and potentially high toxicity^28^,^29^,^30^. In this context CNDs have attracted high interest over the recent years as they share common advantages with QDs, such as high photostability, large two-photon cross-sections^31^ as well as suitability for bio-imaging^6^,^7^,^9^,^15^,^22^. However, in contrast to QDs, CNDs are non-toxic, non-blinking, typically biocompatible and soluble in water^6^,^7^,^9^,^22^. Herein, we report a fast, simple and cost-efficient method based on microwave-assisted hydrothermal precursor carbonization (denoted as MW-hPC), in which, contrary to earlier reports^6^,^8^,^22^,^23^,^32^,^33^,^34^,^35^, only a single synthesis step is necessary to obtain homogenous water-soluble N-CNDs with no need for further purification. Our method involves aqueous solutions of starch (as a carbon source) and TAE buffer (as a nitrogen additive). Starch meets aforementioned requirements since it can be obtained worldwide at low cost with well-defined properties in a wide variation of types and in large quantities without being in competition with food industry. The here-described microwave treatment shortens the reaction time from typically hours^5^,^6^,^15^,^21^,^32^ to several minutes and eliminates steps involving toxic or dangerous materials, whilst being scalable and easy to operate. Furthermore the obtained CNDs are comparatively small (C-CNDs (2.0 ± 0.24) nm; N-CNDs (2.4 ± 0.25) nm) and show narrow size distributions (see Fig. S1). The N-CNDs prepared using our method have good solubility in water, high colloidal stability and high photostability either in solution or as dried white powder (Fig. 1) making them excellent fluorescent materials for e.g. bio-imaging, chemical sensing and optoelectronic devices like LEDs.

## Results

**Figure 1:**
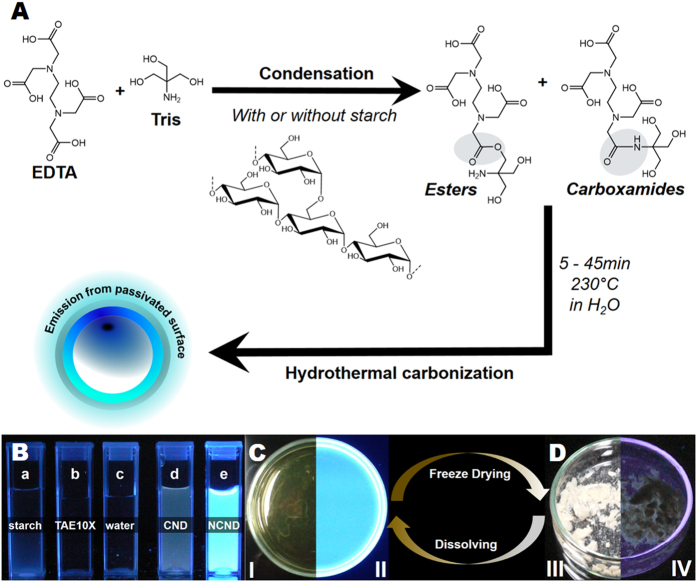
(**A**) The preparation of starch-derived fluorescent C-CNDs and N-CNDs. (**B**) Starting materials: (a) starch, (b) TAE-buffer 10X, (c) water and (d) C-CND and (e) N-CND 1 under UV-light (312 nm). A comparison between N-CND 1 emission in solution (**C**) and as dried powder (**D**) under white light (I; III) and under 312 nm UV-light (II; IV), respectively.

During hydrolysis starch undergoes depolymerisation, which leads to the formation of two main products: *α*-D-glucose and organic acids (e.g. acetic acid)^36^,^37^. To gain a fundamental understanding of the above mentioned process, the influence of starch type and reaction time on the CND formation was examined using UV-Vis absorption as well as PL steady-state and PLQY measurements. Throughout the ongoing reaction the initially transparent colorless liquid sample turned into a yellow clear solution after 15 min, and became deep orange to brown as the reaction progressed. After 45 min blue emission could be observed for all samples (see Fig. 1B and Table S1) when exposed to UV-light (*λ*_max_ = 312 nm). After freeze-drying for three days the former dark yellow solutions turned into a white powder. The powder did not exhibit any PL under UV-light (Figs 1C,D and S2). PL properties recurred (Figs 1C,D and S2) upon dissolving the white carbon powder in polar solvents (e.g. water, ethanol and acetone).

### Synthesis and Characterization of the Starch Derived Carbon Nanodots (C-CND)

#### The influence of reaction time on the C-CND formation

**Figure 2:**
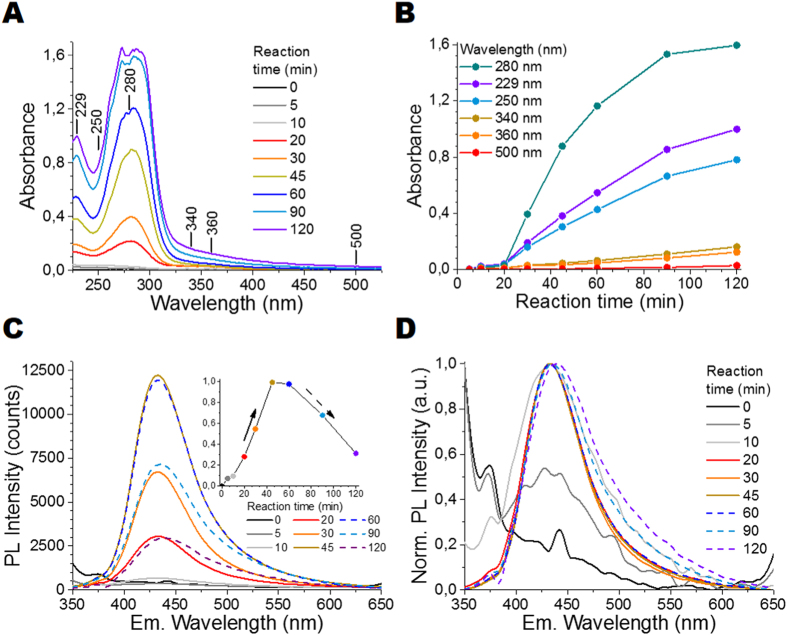
Effect of reaction time on the optical properties of C-CNDs: (**A**) UV-Vis absorption spectra; (**B**) Absorbance at six selected wavelengths as a function of reaction time; (**C**) PL emission spectra for different reaction times under excitation at 340 nm (OD 0.1), the inset is the PL Intensity as a function of reaction time; and (**D**) their corresponding normalized PL emission spectra.

The influence of reaction time on the C-CND formation is illustrated in Fig. 2. In Fig. 2A, UV-Vis absorption spectra exhibit two strong separate absorption bands: the first with the peak at around 250 nm (Peak 1) is assigned to the *π*-π* transition within C=C bonds in aromatic sp2 domains. The band with the peak at around 300 nm to 400 nm (Peak 2) can presumably be attributed to n-π* transition within the C=O bonds, located either on the C-CND surface or within the C-CND core^38^. Conjugated *π*-systems, which also absorb in the 300 nm to 400 nm region, can be excluded as XRD and Raman measurements (see Fig. S3) show that CNDs consist mainly of amorphous carbon (up to 99.9%). The absorption spectra were taken for samples at different reaction times of a C-CND precursor solution (at 200 °C) with fixed starch concentration (undefined starch: 1 mg/mL). The intensities of Peak 1 and Peak 2 increase with the reaction time due to increasing CND concentration, reaching their maximum after 120 min (Fig. 2B). The corresponding PL and normalized PL emission spectra collected with excitation at 340 nm are shown in Fig. 2C,D, respectively. After 5 min reaction time no clear emission band can be observed as the C-CND formation has just started. After 10 min, a weak emission band at around (433 ± 2) nm with an average fluorescence lifetime of 2.22 ns is observed. The standard deviation (±2 nm) was determined based on fluorescence measurements executed for ten C-CND samples obtained from ten synthesis attempts (N = 10), which indicates high reproducibility of the presented synthesis method. PL emission intensity increases as the reaction time increases, reaching its maximum after 45 min (inset in Fig. 2C). Subsequently, the overall PL intensity decreases (dashed line) and a slight emission red-shift from 433 nm to 438 nm can be observed simultaneously. Additionally, a loss in colloidal stability occurs after 120 min. The decreasing PL intensities and subsequent shift result either from the destruction of stabilizing surface groups and/or C-CND aggregation e.g. due to stacking. This is supported by the Iodine tests^39^, which showed a total starch conversion after 45 min (see SOI). Thus, the changes in absorption spectra at reaction times longer than 45 min can only result from further changes in the molecular structure or assembly of C-CNDs. In summary, the C-CNDs carbon core (where absorption presumably takes place)^40^ stays intact and grows, while the emissive surface states are quenched. Further PL measurements demonstrated that the PLQY was below 1% and it was independent of the reaction time. The same measurements executed for starch concentrations at 0.14 mg/mL and 0.28 mg/mL led to similar results, but much lower reaction rates (see Fig. S4). The results show that the carbonization of starch by hydrothermal treatment is probably an autocatalysis process. This autocatalysis is most likely based on the formation of carboxylic acids, which increase the hydrolysis rate significantly. The overall yield of C-CNDs obtained out of starch via hydrothermal treatment is around 80% (w/w) (see Table S2). Above results demonstrate that the formation of CNDs is independent of the starch concentration, but most efficient at high starch concentrations due to stronger autocatalysis process, enabling larger amounts of C-CNDs to be synthesized in shorter time.

#### The influence of starch type on the C-CND formation

The effect of starch type on the C-CND PL properties has been investigated and the results are shown in Fig. S5 (see SOI). As it can be seen in Fig. S5, independently of starch type the C-CND show PL emission peaking at around 434 nm when excited at 340 nm. The PLQY for the un-doped C-CND solutions was found to be below 1%.

Like all natural products, starch undergoes seasonal changes and particularly the amylose/amylopectin ratio is influenced by plant species (Fig. S6) and area of plant cultivation, which could influence the CND formation. Noticeably, the optical properties stayed unaffected for different starch types, even when modified starch was used. Independently of seasonal changes and origin starch provides C-CNDs with highly reproducible PL properties. Nearly any commercial starch currently available on the market can be used to produce CNDs. Additionally, in comparison to other methods used to prepare CNDs, the hydrothermal starch decomposition leads to barely any impurities or by-products, and thus starch can be considered as superior over other carbon sources.

### Synthesis and Characterization of Nitrogen-Doped Carbon Nanodots (N-CND)

**Figure 3:**
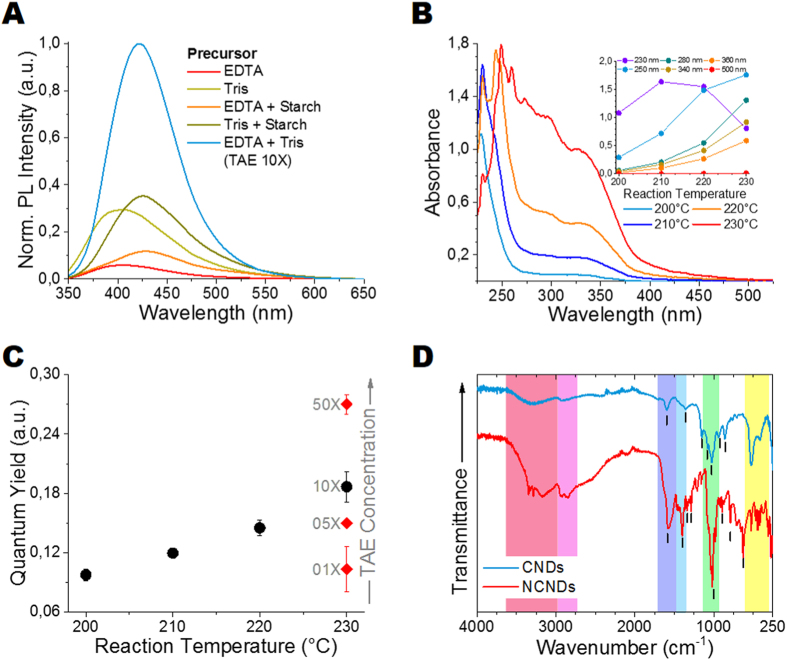
Effect of different N-additive combinations on the (**A**) PL emission spectra (Exc. 340 nm) of samples prepared at 45 min and 230 °C normalized to PLQY. Effect of reaction temperature on N-CNDs prepared from TAE-buffer 10X (EDTA + Tris) using a reaction time of 45 min, (**B**) UV-Vis absorption spectra, and the inset is the absorbance at six selected wavelength as a function of reaction temperature; and (**C**) PLQY at 340 nm. (**D**) FTIR spectra of the as-synthesized carbon nanodots (CND) and nitrogen-doped carbon nanodots (N-CND). TAE-buffer: Tris-acetate-EDTA buffer, EDTA: ethylenediaminetetraacetic acid, Tris: tris(hydroxymethyl)aminomethane.

Nitrogen-doped carbon nanodots (N-CNDs) have emerged to be complementary to C-CNDs due to their high PLQYs^33^,^34^,^41^,^42^,^43^, making them promising luminescent materials for various applications. Here, we study the effect of nitrogen (N) additives, i.e. ethylenediaminetetraacetic acid (EDTA); tris(hydroxymethyl)aminomethane (Tris) and a combination of both (TAE-buffer) on the photophysical properties of CNDs. The impact of reaction temperature on the PL properties is also evaluated, as it was shown that higher reaction temperatures can lead to improved nitrogen incorporation into carbon structures^25^,^34^. To the best of our knowledge, the combined use of EDTA and Tris as N-additives in CNDs synthesis has not been reported to date. The physical properties (e.g. molar mass, structure) of the N-additives can be found in Table S3. The normalized PL spectra of N-CNDs are shown in Fig. 3A. The experimental conditions for all samples, 45 min at 230 °C, were the same. While PL emission could be observed in all cases, the PL, the PL emission maximum and the overall quantum yield varies considerably depending on N-additive combination (see Table 1). Compared to pure N-additive derived samples, the addition of starch leads to a strong red-shift in the PL emission, but has nearly any effect on the overall PLQY. While low PLQYs were observed for the EDTA derived samples (see Table 1), much higher PLQYs were obtained for samples derived from Tris.

The highest PLQYs were observed for the combination of Tris and EDTA as TAE-buffer (19% for 10X TAE buffer and 28% for 50X TAE buffer, see below) without starch addition. The 10X TAE buffer contains acetic acid (200 mM), EDTA (10 mM) and Tris (400 mM), and the TAE based CNDs are here referred to as N-CND 1 (starch in TAE) and N-CND 2 (TAE only). The overall yield of N-CND 2 was calculated to be ~80% (w/w), matching the results from previous CNDs^23^,^32^,^34^ (see Table S2).

#### The influence of reaction temperature on the N-CND formation

The influence of reaction temperature on the UV-Vis absorption spectra and PLQY for N-CND 2 is shown in Fig. 3B,C; for normalized PL emission spectra see Fig. S7. As presented in Fig. 3B, the UV-Vis absorption spectra are composed of three strong absorption bands: two peaks around 270 nm–320 nm and 320 nm–400 nm can be attributed to n-π* transitions in C=O bonds, while the peak at around 225 nm is assigned to C-N bonds located either on the N-CND surface or in the N-CND core. Noticeably, the typical peak at around 275 nm (see Fig. 2A) deriving from *π*-π* transition in C=C bonds in aromatic sp2 domains, couldn’t be observed anymore. This is either due to high C-N signal intensities or missing aromatic sp2 domains in N-CNDs. With increasing reaction temperature the total absorption increases drastically, while the general shape of the spectra remains virtually unaffected. The normalized PL emission spectra (see Fig. S7) show that the emission band, despite different reaction temperatures, is always positioned at around 419 nm (average fluorescence lifetime: 13.02 ns) when excited at 340 nm. Only a slight increase in FWHM, from 69 nm at 200 °C to 78 nm at 230 °C was noticed. This result supports the notion that for CNDs the nature of the surface states determines the emission wavelength. The highest PLQYs for N-CND 1 of 17% (10X TAE + starch) and for N-CND 2 of 28% (50X TAE) were obtained for a reaction temperature of 230 °C, which is lower than the recently reported PLQY for an alginate/tryptophan mixture (47.9%)^32^, but higher than the PLQY values previously reported for N-CNDs obtained from citric acid and urea (14%)^35^, EDTA (17.5%)^34^, and waste chicken eggshell and urea (7.8%)^23^. The strong linearity between PLQY and synthesis temperature (Fig. 3C) suggests that higher PLQYs could be achieved with much higher reaction temperatures^6^. This can be explained by the formation of favourable N-doping architecture^25^ and/or higher surface to volume ratios without a clear correlation between nitrogen content of the precursors and the resulting N-CNDs^25^. Based on the above mentioned results, the reason for the PL enhancement in N-CNDs, compared to un-doped CNDs cannot solely be attributed to nitrogen groups on the N-CNDs surface. Both EDTA and Tris have nitrogen groups, and therefore, are potential N-additives on their own, but only when combined they reach their full potential. If TAE-buffer is used as precursor, most likely esters (via condensation reaction) and carboxamides (via potential CONH2 bonding) are formed between EDTA, Tris and starch (if present), as shown in Fig. 1. Therefore much more nitrogen groups are incorporated into the precursor network and thus into the N-CND surface and core, leading to high PLQY for the as-synthesized N-CNDs. The functional groups within our C-CNDs and N-CNDs were characterized using FT-IR spectroscopy (see Fig. 3D), confirming successful nitrogen bonding. For C-CNDs the broad peak at around 3290 cm^−1^ can be attributed to OH stretching vibrations, indicating the presence of carboxyl and hydroxyl groups, which enable good water solubility and long-term stability. The broad absorption band present at 2700 cm^−1^ (C-H stretching), 1357 cm^−1^ (rock alkanes), 1150 cm^−1^ (C-H wag) and 1026 cm^−1^ (=C-H bend, alkenes) indicate C-H bonds. The peak at 1597 cm^−1^ can be correlated with C=C double bonds in an aromatic ring, which is backed up by the peak at 860 cm^−1^ for C-H “loop” in aromatic systems. Further carboxyl groups are confirmed by the small peak at 1076 cm^−1^, which can be ascribed to C=O double bonds and the peak at 936 cm^−1^, which is ascribed to O-H bending in carboxylic acids. As shown in Fig. 3D C-CNDs and N-CNDs share some IR bands, thus revealing similar chemical compositions and structures. Noticeably, due to nitrogen doping, new characteristic peaks and a drastically changed fingerprint region emerged. Increasing peak intensity and peak broadening at 3000–3700 cm^−1^ (spectral region marked in red) is due to N-H stretching vibrations appearing in addition to C-H stretching vibrations. The broad bands at 2500–3000 cm^−1^ (aliphatic C-H) are much stronger for N-CNDs than for C-CNDs, due to less aromatic structures in N-CNDs. The strong peaks at 1568 cm^−1^ (N-H bend) and 1398 cm^−1^ (C-N = stretching vibrations) indicate successful nitrogen-doping. The bands at 1337 cm^−1^ (C-N stretch), 1286 cm^−1^ (aromatic amines) and 1020 cm^−1^ (aliphatic amines) further show the diverse character of N-CND structures. Additionally, the strong peaks at 900–625 cm^−1^ can be assigned to N-H wag vibrations. As nitrogen content increases the PLQY of N-CNDs, the logical progression was to increase the content of EDTA and Tris in our synthesis. Both N-additives, EDTA (C_10_H_16_O_8_) and Tris (C_4_H_11_NO_3_), can serve also as carbon sources; especially if used at high concentrations. Therefore, we synthesized N-CNDs without using starch as a carbon source in the precursor solutions.

The elemental composition of C-CNDs and N-CND 2 was analyzed by energy-dispersive X-ray spectroscopy (EDX) as shown in Fig. S8 (see SOI). The as-synthesized C-CNDs consist mostly of C- and O-atoms (56.6 At.%; 40.9 At.%) with minor traces of nitrogen (1.27 At.%) and sodium chloride (Na 0.46 At.%; Cl 0.77 At.%). While C-CNDs contain only traces of nitrogen, the nitrogen content in TAE derived N-CND 2 (10.93 At.%) is nine times higher. The successful nitrogen doping is accompanied by a decrease in both carbon- and oxygen-content (49.98%; 39.07%), indicating that nitrogen is incorporated both into the N-CND surface and core. For comparison with previous CNDs see Table S4 (see SOI).

The PL properties of the N-CND-2 samples, derived from different TAE-buffer solutions, by microwave-assisted hydrothermal treatment at 230 °C for 45 min are shown in Fig. S9 (see SOI). When excited at 340 nm, N-CNDs-2 exhibit strong PL emission centered at (418 ± 1) nm independently of the TAE-buffer concentration. This is in contrast to the changes in PLQY, which increases drastically from 9% for 1X TAE to 28% for 50X TAE (see Fig. 3C). To the best of our knowledge these are the highest PLQY values reported for N-CNDs derived from microwave-assisted treatment prepared without further surface functionalization/passivation. An influence of TAE concentration on the UV-Vis absorption is shown in the Fig. S9. For the samples obtained with 10X and 50X TAE-buffer a strong increase in the absorption at 230 nm was observed. As the peak at around 230 nm is ascribed to the presence of C-N bonds a further increase in absorption with further nitrogen doping can be assumed. The normalized UV-Vis absorption spectra (see Fig. S10) reveal that in N-CND 2, in contrast to N-CND 1 (starch + TAE), the peak at 230 nm is much stronger than the peaks for the n-π* transition of the C=O bonds (290 nm, 350 nm) and the peak for *π*-π* transition of C=C bonds in aromatic sp2 domains (260 nm). This suggests that N-CND 2 have a different molecular structure than N-CND 1, despite their similar PL emission properties. This indicates again, that the nature of the surface states determines the emission wavelength of CNDs. This is confirmed by the excitation vs emission wavelength measurements (Fig. S11) which show that N-CNDs, despite their narrow size distributions (Fig. S1), follow selective luminescence behaviour, as described in the literature^1^,^7^,^20^. While the PL emission maximum shows a clear excitation wavelength dependency, the FWHM stays nearly the same.

## Discussion

We present a new reliable, one-step CND synthesis based on hydrothermal treatment of natural precursors, with a low environmental footprint and at reasonable costs. Three different CNDs, with a high yield of ~80% (w/w), have been prepared using three different sets of starting materials, referred to as C-CND (starch), N-CND 1 (starch in TAE) and N-CND 2 (TAE only). The as-prepared CNDs are highly homogenous in their photoluminescence properties and ready to use without need for further purification, modification or surface passivation agents (e.g. PEG). The CNDs are comparatively small (C-CND: (2.0 ± 0.24) nm; N-CNDs (2.4 ± 0.25) nm) with narrow size distributions (Fig. S1); are stable over a long period of time, either in solution or as freeze dried powder and maintain their photoluminescence properties when re-dispersed in solution. Depending on starting material, we obtained CNDs with PL quantum yield (PLQY) in the range of <1–28%. For the first time we also addressed the following issues: (1) the influence of reaction time and starch type on C-CND formation, (2) the stability of CNDs over time, both in solution and as dry powder. We also focus on studying the change in photophysical properties (absorption and PL emission spectra as well as PLQY) resulting from the conversion of CND solution into dry white to slightly brown carbon powder and back into the solution. In the present work we have studied the influence of: (a) precursor concentration, (b) type of additives and (c) reaction time on the photophysical properties (absorption and PL emission spectra as well as PLQY) of CNDs. The as-prepared N-CNDs exhibit well-defined, reproducible and favourable photoluminescence (PL) properties with narrow emission bands (approx. 70 nm full width at half maximum (FWHM)), high PLQY (up to 28%), stable emission under a wide range of pH values and long-term stability (at least one year). We believe that our findings provide new ways for the preparation of excellent and well defined CNDs in less reaction time and at reasonable costs, thereby paving the way for future large-scale applications.

## Methods

### Reagents and Materials

Potato starch (20 wt% amylose); wheat starch (28 wt% amylose); corn starch wild type (approx. 27% amylose); waxy corn starch (76 wt% amylose) and low amylose corn starch (0–1 wt% amylose) and TAE buffer were purchased from Sigma-Aldrich Chemie GmbH (Munich, Germany). Modified Starches: Alimentamyl 2002 (distarch phosphate); Amylex 20/20 (depolymerized potato starch); Fibraffin K105 (cationic starch ether); Licocat P (quaternary cationic starch solution); Sobocat (quaternary starch ether); Sobotex 5305 NN (starch acetate) and Sobotex CM (starch ether) were provided by Südstärke GmbH (Schrobenhausen, Germany). The undefined starch was purchased in a supermarket in Germany. Double deionized water from a Milli-Q water system (Merck Millipore, Billerica, MA, USA) was used throughout all experiments.

### Synthesis of carbon nanodots (CNDs)

In a typical CND synthesis 0.014 mg/mL to 10 mg/mL of the respective starch were dissolved in 4 mL of deionized water (C-CND) or 4 mL of TAE buffer (N-CND 1) at room temperature (RT) for several minutes. To obtain type 2 photoluminescent nitrogen-doped CNDs (N-CND 2) the precursor solutions consisted solely of pure TAE buffer at various concentrations (1X to 50X), all originating from a 50X stock solution containing 242 g Tris base (2 M), 57.1 mL glacial acetic acid (1 M) and 14.612 g of EDTA (50 mM) dissolved in 1 L of water. The pH values of the starting solutions were set to 7.0 and 8.0 for C-CND and N-CND-synthesis, respectively. The as-prepared precursor solutions were then transferred into a pressure resistant glass vial and heated up to 200–230 °C for 5–120 min with a Monowave 300 synthesis microwave oven (Anton Paar GmbH, Graz, Austria). After cooling down to RT, the resulting clear light yellow to brown aqueous solutions needed no further purification and were ready for further characterization. In order to verify total starch conversion, 0.5 mL of the obtained solutions (C-CND; N-CND 1) were added to 50 *μ*L of Lugol’s Iodine solution. Finally, the CND-solutions were freeze dried and stored at room temperature for further measurements.

### Characterization

The UV-Vis absorption spectra were collected on a Perkin-Elmer Lambda 750 and a Nanodrop200c spectrophotometer (Thermo Fisher Scientific Inc., Waltham, MA, USA). Emission spectra were collected using a FluoroMax-P fluorescence spectrometer (HORIBA Jobin Yvon GmbH, München, Germany). PL quantum yield (PLQY) measurements were carried out on a modular C9920-02/-03 (Hamamatsu Photonics, Japan) including an integrating sphere, a PMA-12 Photonic Multi-Channel-Analyser C10027-01 (Hamamatsu Photonics, Japan) as detector and a 150 W/CW Xe lamp L10092 (Hamamatsu Photonics, Japan) as excitation source. Samples were placed in a 1.0 × 1.0 cm quartz cuvette and measurement data was collected, processed, visualized and analysed automatically by built-in software. Time-correlated single photon counting measurements were done on a FLS920 Fluorescence spectrophotometer (Edinburgh Instruments, UK). The samples were measured in a 90° setup using a white light source (SC-400-PP supercontinuum-source, Fianium: 0.5–20 MHz, 400 nm < l < 24000 nm, pulse width: ca. 30 ps) as excitation source and a Multi-Channel-Plate (ELDY EM1–132/300, Europhoton GmbH, Berlin, Germany) as detector. The Fluorescence decays were fitted using the FAST software (Edinburgh Instruments, UK). To avoid filter effects and other effects that might distort the signal readout, the PL emission and PLQY measurements were carried out at an optical density (OD) equal to 0.1 at the corresponding excitation wavelength. Fourier transform infrared (FT-IR) spectra were collected using a Bruker Tensor 27 FT-IR spectrometer (Bruker Optics, Billerica, MA, USA) using diamond in attenuated total reflection (ATR) in the range of 400–4000 cm^−1^.

Elemental analysis was performed on Elementar Vario EL III (Elementar Analysensysteme GmbH, Hanau, Germany). Laser diffraction particles size analysis (SLS) was performed on LS 13 320 Laser Diffraction Particle Size Analyser (Beckman Coulter Inc., Brea, CA, USA). TEM images were recorded on a JEOL JEM 1011 microscope (JEOL Germany GmbH, Freising, Germany) operated at 200 kV with samples prepared on 400 mesh, SiO_2_ coated copper grids (Plano GmbH, Wetzlar, Germany). Evaluation of obtained TEM images (particle recognition; measurement and analysis of corresponding particle sizes) was performed using Pebbles and PebbleJuggler^44^. X-ray diffraction (XRD) patterns of the as-prepared CNDs were recorded on a EMPYREAN diffractometer (PANalytical B.V., Almelo, Netherlands) equipped with CuK*α* (*λ* = 0.15405 nm) radiation at scanning speed of 0.47 °/min in the range from 4° to 70°. X-ray microanalysis was performed on a Zeiss ULTRA55 PLUS scanning electron microscope (Carl Zeiss Microscopy GmbH, Jena, Germany), operating at 5 to 10 kV and a working distance of 10 mm. EDX microanalyses with an acquisition time of 20–30 min and a Quantax 400 (Bruker Corporation, Billerica, MA, USA) as detector have been obtained for both the C-CND and the N-CND 2. The elemental quantitative analysis used an automatic background subtraction and a correction has been used to calculate the elemental composition in weight percents and atomic percents.

## Additional Information

**How to cite this article**: Meiling, T. T. *et al*. White carbon: Fluorescent carbon nanoparticles with tunable quantum yield in a reproducible green synthesis. *Sci. Rep.* **6**, 28557; doi: 10.1038/srep28557 (2016).

## Supplementary Material

Supplementary Information

## Figures and Tables

**Table 1:** The results of PL emission and PLQY for five different N-additive combinations, including N-CNDs.

*λ*_ex_ = 340 nm	Pure EDTA	Pure Tris	EDTA + starch	Tris + starch	Starch in TAE 10X N-CND 1	TAE 10X N-CND 2	TAE 50X N-CND 2
PL emission [nm]	408	408	429	425	419	419	419
PLQY [%]	1	6	2	6	17	19	28
EDTA content [mg/mL]	2.93	–	2.93	–	2.93	2.93	2.93
Tris content [mg/mL]	–	48.5	–	48.5	48.5	48.5	242.5
Starch content [mg/mL]	–	–	48.5	48.5	0.14	–	–

TAE-buffer: Tris-acetate-EDTA buffer, EDTA: ethylenediamine-tetraacetic acid, Tris: tris(hydroxymethyl)aminomethane; TAE 10X contains: acetic acid (200 mM), EDTA (10 mM) and Tris (400 mM).
