# Supplementary material for: White carbon: Fluorescent carbon nanoparticles with tunable quantum yield in a reproducible green synthesis
Source: Sci Rep. 2016 Jun 23;6:28557. doi: 10.1038/srep28557 (PMC4917870; doi:10.1038/srep28557)
Supplement: Supplementary Information [file srep28557-s1.doc]

Supporting Information

White carbon: Fluorescent carbon nanoparticles with tunable quantum yield in a reproducible green synthesis.

T.T. Meiling*^1^*, P. J. Cywinski*^2,3^** and I. Bald*^1,4^**

*^1^Physical Chemistry, Institute of Chemistry, University of Potsdam, Karl-Liebknecht-Str. 24-25, 14476 Potsdam-Golm, Germany*

*^2^Functional Materials and Devices, Fraunhofer Institute for Applied Polymer Research, Geiselbergstr. 69, 14476 Potsdam-Golm, Germany*

*^3^Coordination Complexes and Functional Materials, Institute of Physical Chemistry, Polish Academy of Sciences, Kasprzaka 44/52, 01-224 Warsaw, Poland*

*^4^BAM, Federal Institute of Material Research and Testing, Richard-Willstätter-Straße 11, 12489 Berlin, Germany*

* Corresponding authors:

Prof. Dr. Ilko Bald: E-mail: [bald@uni-potsdam.de](mailto:bald@uni-potsdam.de), Phone: +49 331 977-5238, Fax: +49 331 977-6167

Dr. Piotr J. Cywinski: Email: [piotr.cywinski@ichf.edu.pl](mailto:piotr.cywinski@ichf.edu.pl), Phone: +48 22 343-2076, Fax: +48 22 343-3333

***
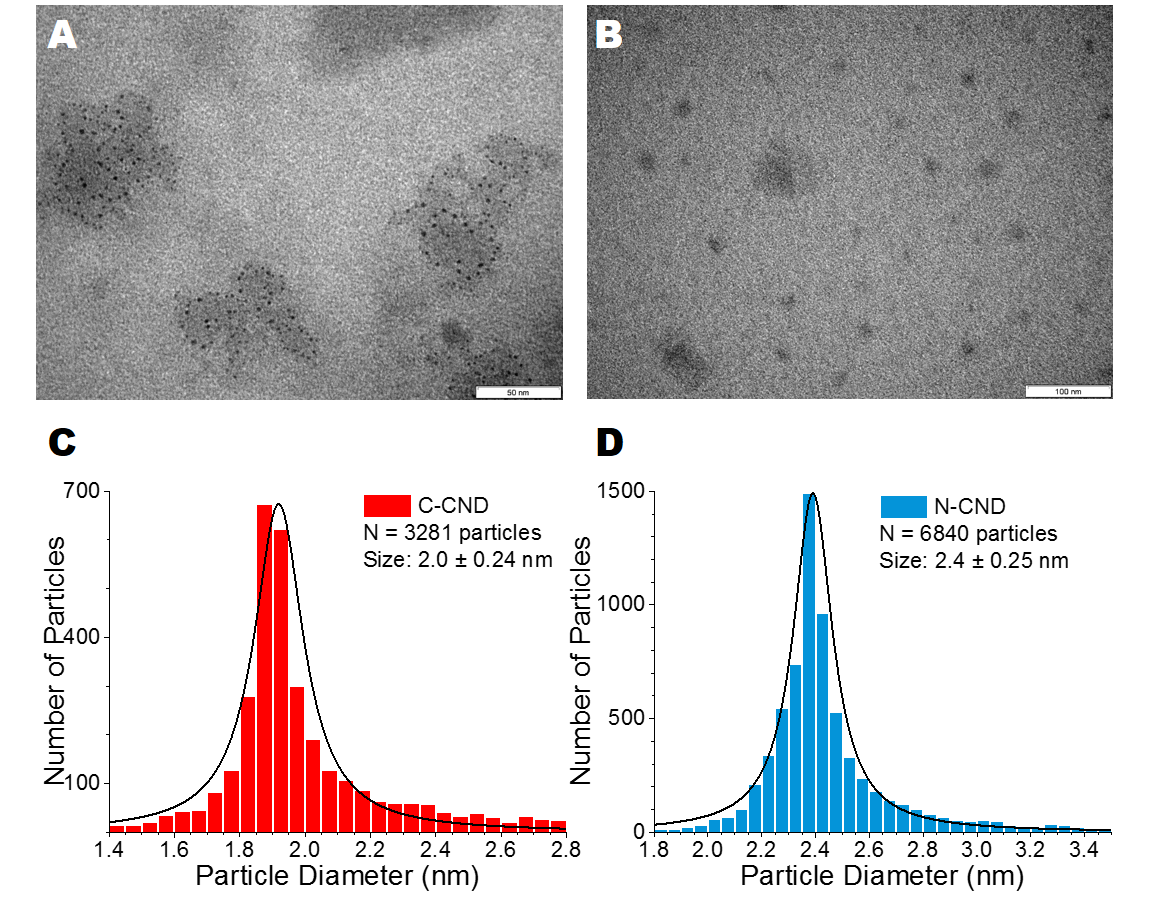
*Figure S1.** TEM images of (**A**) C-CNDs; (**B**) N-CNDs. The corresponding particle size distributions and Gaussian curves obtained from the fitted data, are shown (**C**) C-CND and (**D**) N-CND, respectively. Obtained from multiple Images the average particles sizes are reported at the 99% confidence interval.

**Table S1.** The results of PL emission, QY and average fluorescence (FL) lifetime for three different kinds of CNDs synthesized in our study.

| Sample | Composition of precursor | PL emission (nm) | QY (%) | FL lifetime τ (ns) |
| --- | --- | --- | --- | --- |
| **C-CND** | starch (1 mg/mL) | 433 | >1 | 2.22 |
| **N-CND 1** | starch (0.14 mg/mL) in TAE 10X (EDTA 10mM; Tris 400 mM; acetic acid 200 mM) | 419 | 17 | 13.05 |
| **N-CND 2** | TAE 50X (EDTA 50mM; Tris 2M; acetic acid 1M) | 419 | 28 |


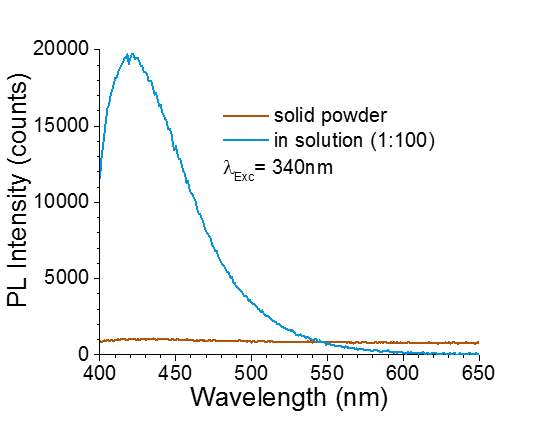


**Figure S2.** PL emission spectra of N-CND as dried solid powder (brown) and the same sample (diluted 1:100) as aqueous solution (blue).


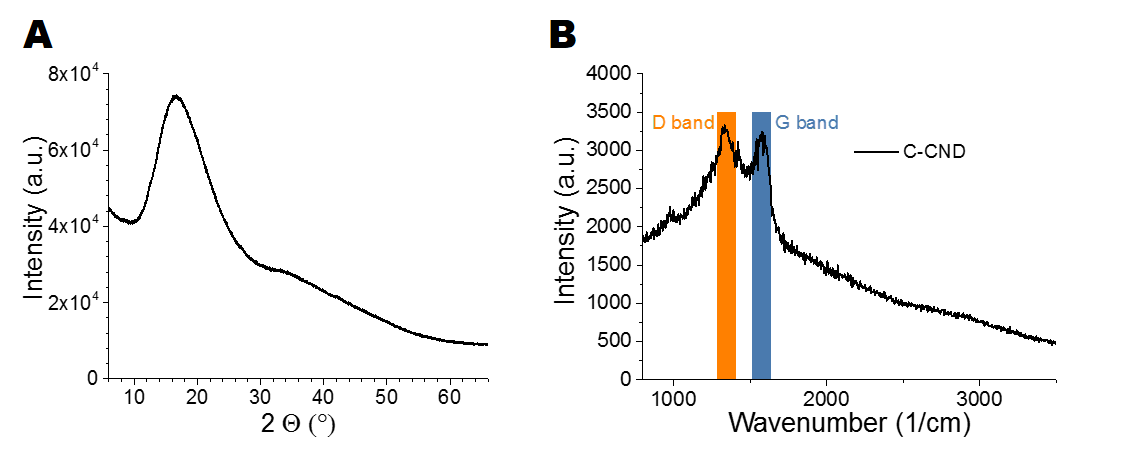


**Figure S3.** Typical (**A**) X-ray diffraction (XRD) pattern and (**B**) Raman spectra of the as prepared CNDs.


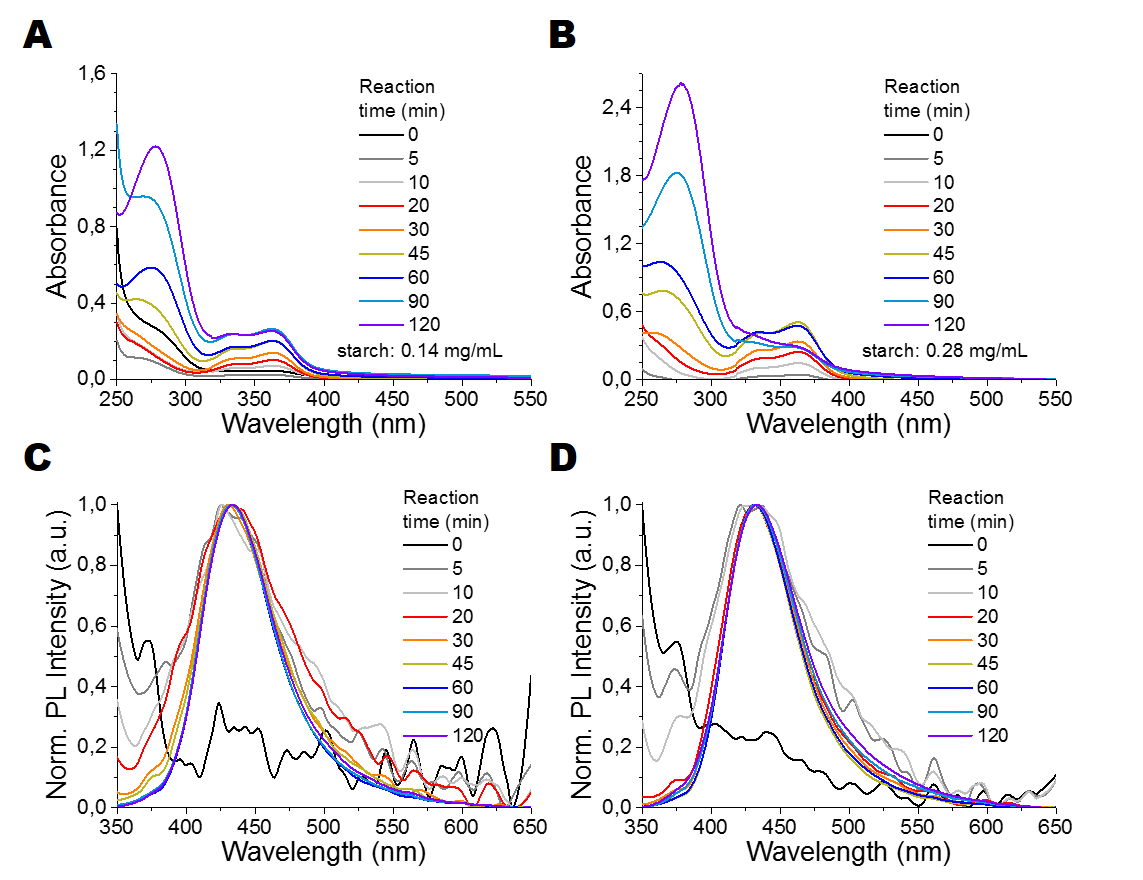


**Figure S4.** Effect of reaction time on the optical properties of C-CNDs for different starch (precursor) concentrations: UV-Vis absorption spectra as a function of reaction time for (**A**) 0.14 mg/mL starch and (**B**) 0.28 mg/mL starch; normalized PL emission spectra for different reaction times under excitation at 340 nm (OD 0.1) for (**C**) 0.14 mg/mL starch and (**D**) 0.28 mg/mL starch.


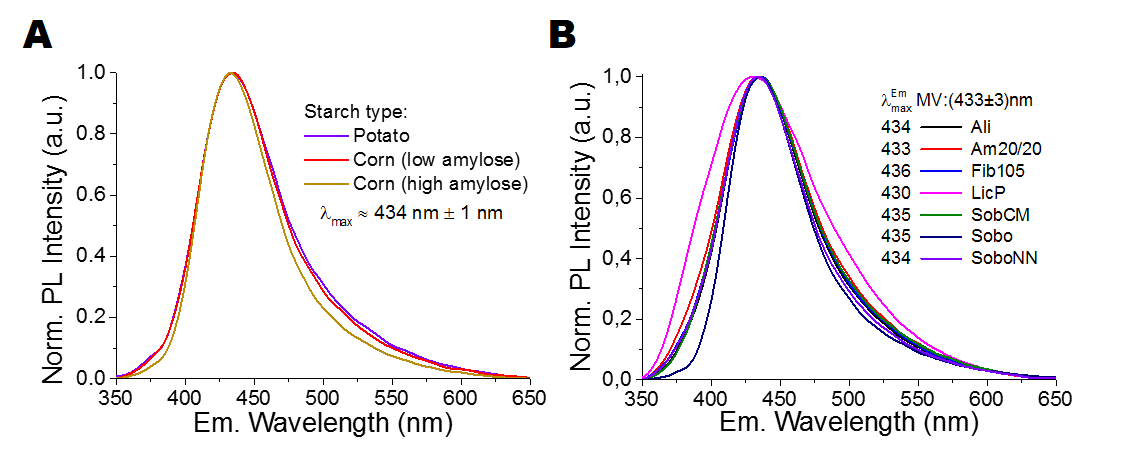


**Figure S5.** Effect of starting material (starch type) on the normalized PL emission spectra of C-CNDs under excitation at 340 nm (OD 0.1): (**A**) Potato starch (20 wt% amylose), low amylose corn starch (0-1 wt% amylose) and waxy corn starch (76 wt% amylose); (**B**) Ali: Alimentamyl 2002 (distarch phosphate), Am20/20: Amylex 20/20 (depolymerized potato starch), Fib105: Fibraffin K105 (cationic starch ether), LicP: Licocat P (quaternary cationic starch solution), Sobo: Sobocat (quaternary starch ether), SoboNN: Sobotex 5305 NN (starch acetate) and SoboCM: Sobotex CM (starch ether). The standard deviation is based on PL measurements executed for all starch types used. The QY for all samples was found to be below 1%.


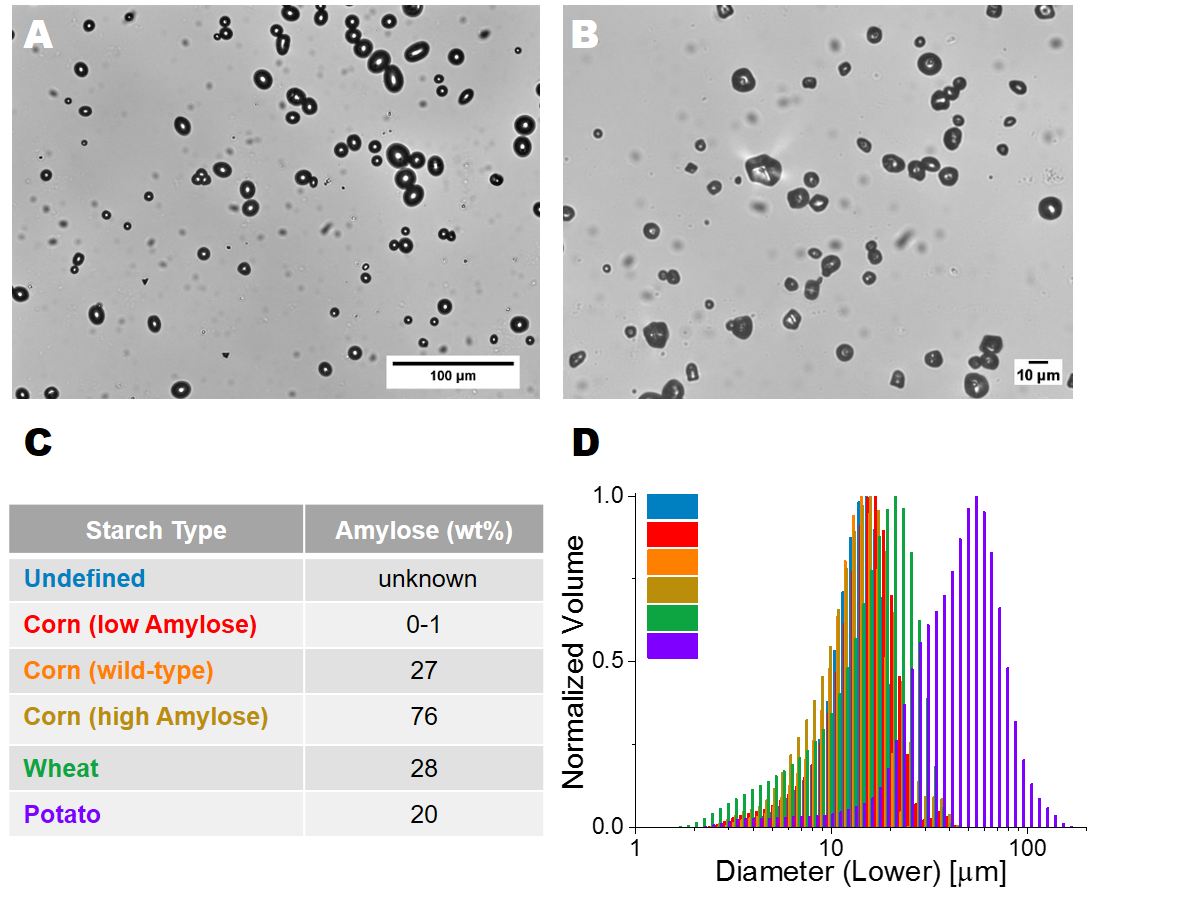


**Figure S6.** Light microscope images of starch particles from (**A**) potato starch and (**B**) corn starch. Amylose content (wt%) for the used natural starches (**C**) and their starch particle size distributions (**D**) obtained by laser diffraction particles size analysis (SLS).

**Table S2.** The yield of CNDs from previous articles.

| **Method** | **Carbon precursor** | **Yield (%)** | **Reference** |
| --- | --- | --- | --- |
| MW-hPC | TAE / starch | ~ 80% | This work |
| Calcination | Chicken eggshell | 81-85% | 1 |
| Hydrothermal | EDTA | 78% | 2 |
| Calcination | Sodium alginate and tryptophan | 62% | 3 |
| Hydrothermal | Citric acid and ethylenediamine | 58% | 4 |
| Organic pyrolysis | Dextrin | 38% | 5 |
| Hydrothermal | Cow milk | 34.5% | 6 |
| Organic pyrolysis | Egg white / egg yolk | 5.96% | 7 |

*MW-hPC: microwave-assisted hydrothermal precursor carbonization

**Table S3.** N-additives used in our study: ethylenediaminetetraacetic acid (EDTA) and tris(hydroxymethyl)aminomethane (Tris).

|  | Chemical formula | Molar mass (g/mol) | Structure | Number of functional groups |
| --- | --- | --- | --- | --- |
| EDTA | C_10_H_16_N_2_O_8_ | 292.24 | 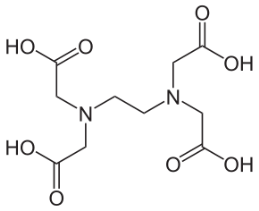 | N_COOH_ = 4 |
| Tris | C_4_H_11_NO_3_ | 121.14 | 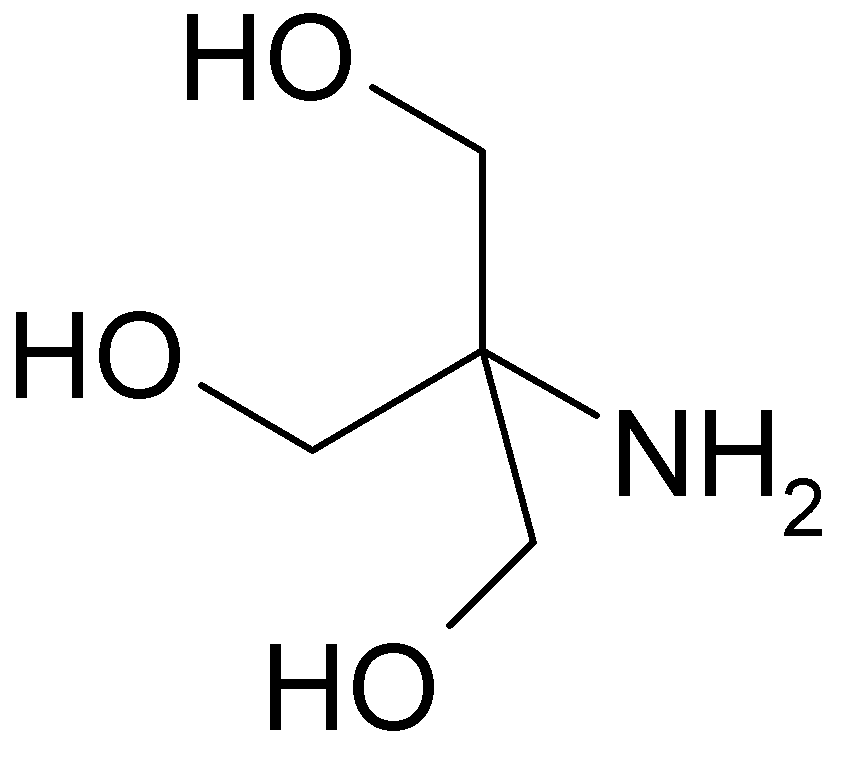 | N_OH_ = 3  N_NH2_ = 1 |

**Table S4.** The quantum yields, elemental content and elemental ratio of CNDs from previous articles.

| **Reference** | **Sample** | **Quantum yield** | **Elemental content (%)** | | | **Element ratio** | |
| --- | --- | --- | --- | --- | --- | --- | --- |
|  |  |  | **C** | **N** | **O** | **N/C** | **O/C** |
| This work | C-CND | < 1% | 56.6 | 1.26 | 40.9 | 0.02 | 0.72 |
|  | N-CND 2 | 28% | 56.35 | 10.57 | 33.07 | 0.19 | 0.59 |
| 1 | NCND 1 | 4.2% | 78.1 | 4.24 | 15.71 | 0.05 | 0.20 |
|  | NCND 2 | 7.8% | 60.0 | 15.4 | 19.4 | 0.26 | 0.32 |
| 2 | - | 17.5% | 61.01 | 6.33 | 28.05 | 0.10 | 0.46 |
| 3 | - | 47.9% | Not given | | | 0.04 | Not given |
| 4 | CD 4 | 17.3% | 70.25 | 10.63 | 11.97 | 0.15 | 0.17 |
|  | CD 3 | 24.7% | 56.6 | 16.13 | 20.5 | 0.28 | 0.36 |
|  | CD 2 | 60.2% | 51.13 | 16.25 | 26.81 | 0.32 | 0.52 |
|  | CD 1 | 75.2% | 48.01 | 33.72 | 13.23 | 0.28 | 0.70 |
| 5 | - | 5% - 9% | Not given | | | | |
| 6 | - | 9.6% | 92.15 | 3.66 | 4.18 | 0.04 | 0.05 |
| 7 | CD_pew_ | 6% | 56.75 | Not given | | | |
|  | CD_pey_ | 8% | 62.42 | Not given | | | |


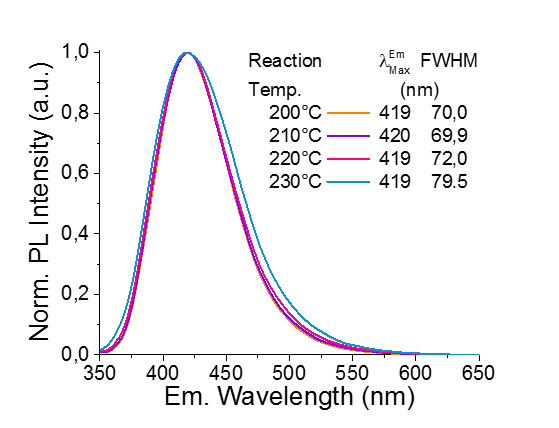


**Figure S7.** Effect of reaction temperature on normalized PL emission spectra under excitation at 340 nm (OD 0.1) of N-CNDs prepared from TAE-buffer 10X using a reaction time of 45 min. TAE-buffer: Tris-acetate-EDTA buffer, EDTA: ethylenediaminetetraacetic acid, Tris: tris(hydroxymethyl)aminomethane; TAE 10X contains: acetic acid (200mM), EDTA (10 mM) and Tris (400mM).


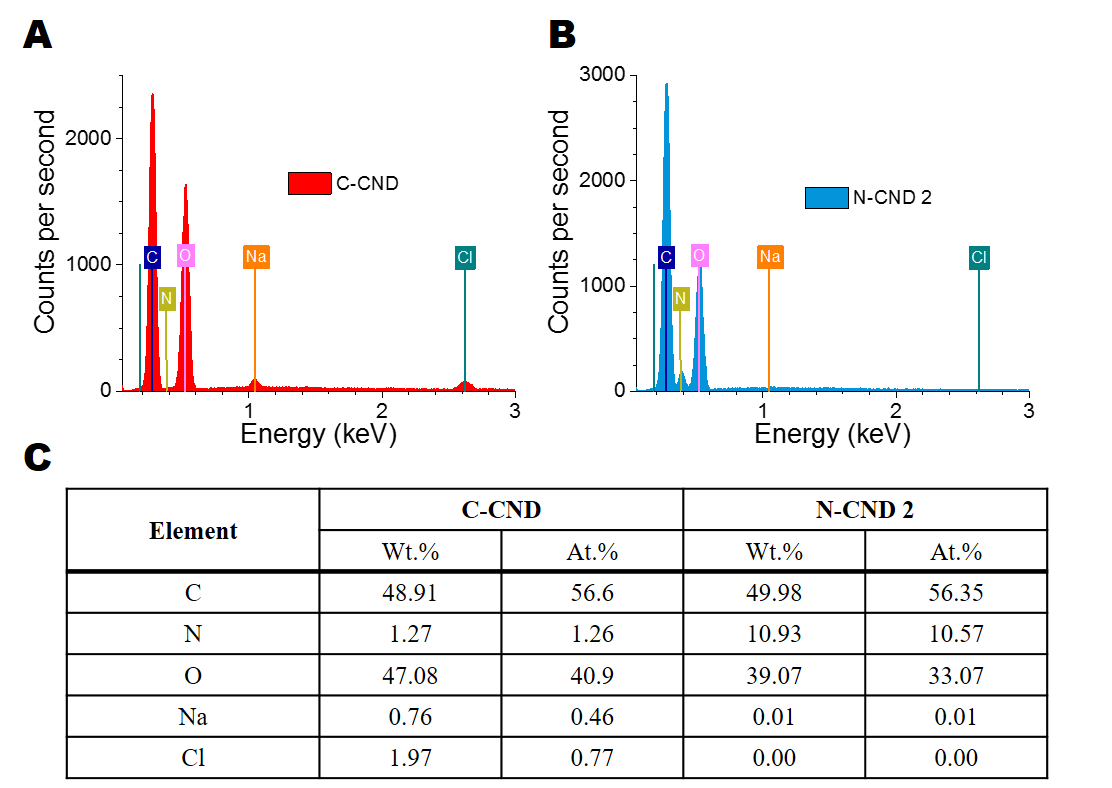


**Figure S8.** Typical EDX spectra of (**A**) C-CND; (**B**) N-CND 2 at 10kV operation voltage and (**C**) the tabulated results of the elemental composition in units of both weight percent (Wt.%) and atomic percent (At.%).


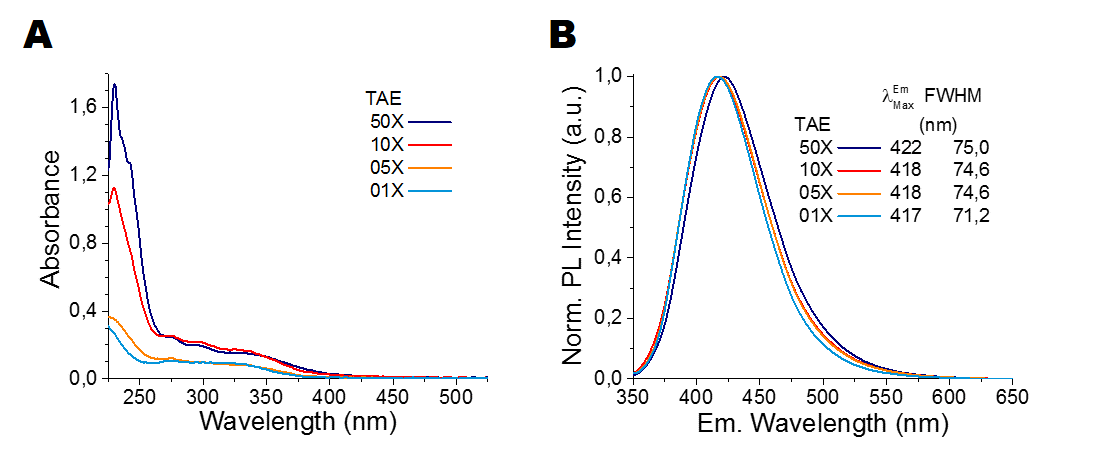


**Figure S9.** Effect of different TAE-buffer (N-additive) concentrations on the (**A**) UV-Vis absorption spectra and (**B**) normalized PL emission spectra under excitation at 340 nm (OD 0.1). TAE-buffer: Tris-acetate-EDTA buffer; EDTA: ethylenediaminetetraacetic acid, Tris: tris(hydroxymethyl)aminomethane; TAE 50X contains: acetic acid (1M), EDTA (50 mM) and Tris (2M).


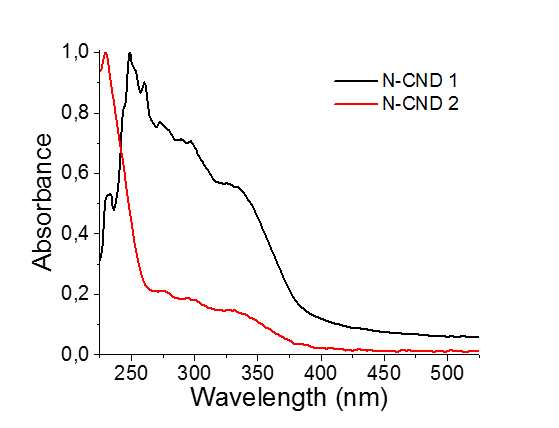


**Figure S10.** Normalized UV-Vis absorption spectra for N-CND 1 (starch in TAE) and N-CND 2 (pure TAE).

**
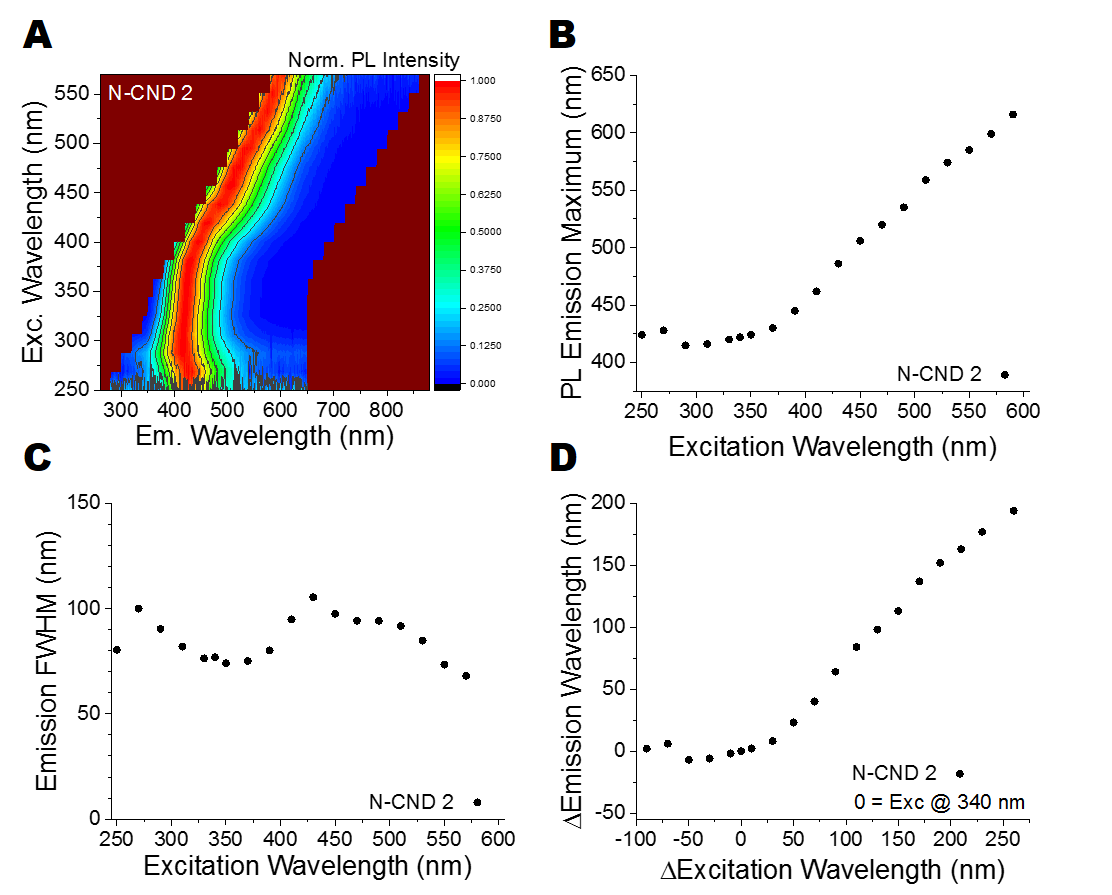
**

**Figure S11.** (**A**) Excitation wavelength vs normalized PL emission and influence of excitation wavelength on PL emission properties for N-CND 2 (TAE 50X) on (**B**) PL emission maximum and (**C**) PL emission full width at half maximum (FWHM). The influence of excitation wavelength shift vs the resulting PL emission wavelength shift (**D**).

**Supplementary References**

1. Ke, Y., Garg, B. & Ling, Y.-C. Waste chicken eggshell as low-cost precursor for efficient synthesis of nitrogen-doped fluorescent carbon nanodots and their multi-functional applications. *RSC Adv.* **4**, 58329–58336 (2014).
2. Shi, Q.-Q. et al. High-yield and high-solubility nitrogen-doped carbon dots: formation, fluorescence mechanism and imaging application. *RSC Adv.* **4**, 1563 (2013).
3. Zhu, X. et al. Nitrogen-doped carbon nanoparticle modulated turn-on fluorescent probes for histidine detection and its imaging in living cells. *Nanoscale* **8**, 2205 – 2211 (2016).
4. Zhu, S. et al. Highly photoluminescent carbon dots for multicolor patterning, sensors, and bioimaging. *Angew. Chem. Int. Ed.* **52**, 3953–3957 (2013).
5. Puvvada, N. et al. Synthesis of biocompatible multicolor luminescent carbon dots for bioimaging applications. *Science and Technology of Advanced Materials* **13**, 045008 (2012).
6. Han, S. et al. Fabrication, gradient extraction and surface polarity-dependent photoluminescence of cow milk-derived carbon dots. *RSC Adv* **4**, 58084–58089 (2014).
7. Wang, J., Wang, C.-F. & Chen, S. Amphiphilic egg-derived carbon dots: Rapid plasma fabrication, pyrolysis process, and multicolor printing patterns. *Angew. Chem. Int. Ed.* **51**, 9297–9301 (2012).
